# Systematic Review on Post-Mortem Protein Alterations: Analysis of Experimental Models and Evaluation of Potential Biomarkers of Time of Death

**DOI:** 10.3390/diagnostics12061490

**Published:** 2022-06-17

**Authors:** Matteo Antonio Sacco, Fabrizio Cordasco, Carmen Scalise, Pietrantonio Ricci, Isabella Aquila

**Affiliations:** Institute of Legal Medicine, Department of Medical and Surgical Sciences, University “Magna Graecia” of Catanzaro, 88100 Catanzaro, Italy; matteoantoniosacco@gmail.com (M.A.S.); cordasco@unicz.it (F.C.); scalisecar@gmail.com (C.S.); ricci@unicz.it (P.R.)

**Keywords:** forensic pathology, time of death, proteins, post-mortem interval

## Abstract

Estimating the post-mortem interval (PMI) is a very complex issue due to numerous variables that may affect the calculation. Several authors have investigated the quantitative and qualitative variations of protein expression on post-mortem biological samples in certain time intervals, both in animals and in humans. However, the literature data are very numerous and often inhomogeneous, with different models, tissues and proteins evaluated, such that the practical application of these methods is limited to date. The aim of this paper was to offer an organic view of the state of the art about post-mortem protein alterations for the calculation of PMI through the analysis of the various experimental models proposed. The purpose was to investigate the validity of some proteins as “molecular clocks” candidates, focusing on the evidence obtained in the early, intermediate and late post-mortem interval. This study demonstrates how the study of post-mortem protein alterations may be useful for estimating the PMI, although there are still technical limits, especially in the experimental models performed on humans. We suggest a protocol to homogenize the study of future experimental models, with a view to the next concrete application of these methods also at the crime scene.

## 1. Introduction

In forensic pathology, estimating the post-mortem interval (PMI) is a real challenge. The calculation is generally performed through the comparison of various parameters, such as cadaveric rigidity, body temperature and hypostases [1]. In addition, diagnosis is supported by the analysis of the transformative phenomena of the corpse, post-mortem ocular alterations and by circumstantial data [2,3]. The accuracy of the PMI calculated with these parameters is a function of the time elapsed since death. Therefore, the longer the time elapsed since death, the more approximate the calculated time range will be. Furthermore, the PMI is influenced by many variables such as ambient temperature, humidity, ventilation, body mass and body temperature at the time of death, which can affect the validity of the calculation. For all these reasons, the forensic pathologist can encounter concrete difficulties, especially when he must backdate the time of death in complex crime scenes characterized, for example, by advanced transformative phenomena in homicides, so that a precise estimation is mandatory. Recently, scientific research has focused on the application of forensic biochemistry as a useful support to improve the PMI estimation [4,5,6,7,8]. Several studies have shown the concrete possibility of an approach with protein analysis to reduce the approximation of the calculated range. In this context, the scientific community has proposed several studies on post-mortem protein expression and on the timing of protein degradation. Numerous experimental models have been proposed, with notable differences regarding the sample examined, the biological matrix analyzed and the methods used. The results obtained are so diversified that it is, therefore, difficult to apply them on the scene in a practical way. In order to offer a harmonious view of the state of the art, we present a literature review on the papers published in the last 20 years until 2021 that have analyzed the variations of proteins in the post-mortem. The review aimed to examine protein alterations after death by comparing the methodologies used and the results obtained. The aim of authors was to evaluate, through an organic analysis, which are the main proteins studied, analyzing their pattern variations after death and discussing the limits in this area of research. This review may prove useful to deepen the study of candidate proteins for “molecular clock” through increasingly precise human experimental models, with a view to the next practical application of these technologies in cases of judicial interest.

## 2. Materials and Methods

The literature review was carried out using the PubMed NCBI and SCOPUS databases. Papers published in the last 20 years were evaluated, i.e., between January 2001 and 2021.

The following key words were used: (“time of death” OR “postmortem interval” OR “post mortem interval” OR post-mortem interval) AND (“forensic” OR “autopsy” or “necropsy” OR “legal medicine”) AND (“protein” OR “proteins” OR “proteic” OR “proteomic” OR “proteomics”).

The articles were selected on the condition that they met the following criteria:

- Quantitative and/or qualitative post-mortem evaluation of proteins on animal or human tissues;
- English language;
- Year of publication from 2001–2021.

The articles were excluded by title, abstract or full text due to the lack of agreement of the works with our inclusion criteria [9].

**Figure 1:**
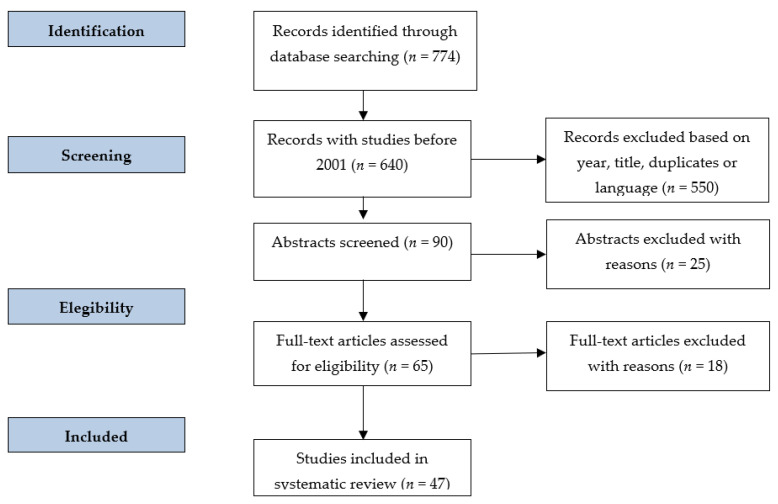
Algorithm followed for the selection of papers on PubMed and SCOPUS databases.

The review included a screening phase of the research, with the exclusion of duplicates. Subsequently, the papers that indicated in the title the evaluation of proteins in the post-mortem were selected for the study of the abstract. The next phase involved a further screening on the basis of the full texts (Figure 1).

The research evaluated the year of publication, sample (human or animal), tissue, methods, marker investigated, PMI investigated and results through the processing of tables. In order to facilitate the study, the table for animal models has been differentiated from that for human studies.

## 3. Results

### 3.1. Database Searching

The inclusion of keywords in the databases provided a total of 774 papers. Of these, papers published before 2001, duplicates and non-English works were excluded. In total, 90 works were screened, of which 65 were chosen for the full-text reading and 47 were included in the review. Of the selected papers, 19 papers (40.4%) concerned experimental animal models and 28 studies (59.5%) concerned human models.

### 3.2. Analysis on Animal Samples

#### 3.2.1. Analysis of Experimental Animal Models

The following animals were used in the studies reviewed: pigs (36.8% of animal studies), rats (36.8%), mice (21%), fish (5.2%) and cattle (5.2%). The number of cases included a minimum of 2 and a maximum of 90 animals per study [10,11,12,13,14,15,16,17,18,19,20,21,22,23,24,25,26,27,28].

#### 3.2.2. Analysis of Biological Animal Samples

The biological samples in the animal studies were: skeletal muscle tissue (47.3% of the animal studies), myocardial tissue (15.7%), bone (15.7%), kidney (15.7%), blood (10.5%), brain (10.5%), lung (10.5%), gastrointestinal tract (5.2%), liver (5.2%), spleen (5.2%) and testes (5.2%).

#### 3.2.3. PMI Examined in Animal Studies

The PMI assessed included a minimum time equal to 0, that is the exact moment of death, and a maximum time of 24 months. The analysis of time zero (78.9% of animal studies) was possible because the experimental models involved the study of markers from sacrificed animals starting from the moment of death and then at different time intervals.

In particular, 16 studies (84.2%) investigated the early PMI (interval 0–24 h from death), 16 studies (84.2%) included the intermediate PMI (time between 1 day–1 month) and 2 studies (10.5%) evaluated the late PMI (1 month–2 years).

#### 3.2.4. Methods Used in Animal Studies

The most used method was Western blotting (63.1%), followed by liquid chromatography–mass spectrometry (LC-MS/MS) (15.7%), ELISA (10.5%) and immunohistochemistry (10.5%). Other methods were used in 21% of animal studies.

#### 3.2.5. Results Obtained in Animal Studies

In 18 studies (94.7%) involving animal models, the variation of at least one protein was observed. Only in one study was there no correlation between PMI and markers evaluated.

The variations obtained were, in most cases, related to protein degradation or decrease (78.9% of studies), in five studies (26.3%) the stability of the examined markers was demonstrated and in three studies (15.7%) there was an increase in the marker (Table 1).

### 3.3. Analysis on Human Samples

#### 3.3.1. Analysis of Human Experimental Models

The analysis of human models involved a minimum of 2 cases and a maximum of 164 cases per study [15,17,29,30,31,32,33,34,35,36,37,38,39,40,41,42,43,44,45,46,47,48,49,50,51,52,53,54,55].

#### 3.3.2. Analysis of Human Biological Samples

The biological samples in the animal studies were: skeletal muscle tissue (25% of the human studies), myocardial tissue (21.4%), brain (21.4%), bone (10.7%), lung (10.7%), blood (7.1%), liver (7.1%), kidney (7.1%), pancreas (7.1%), gingival tissue (7.1%), cerebrospinal fluid (3.5%), thyroid (3.5%) and urine (3.5%).

#### 3.3.3. PMI Examined in Human Studies

The PMI assessed included a minimum time of 0 and a maximum time of 20 years. In the case of human models, time 0 is not to be considered as the exact moment of death but as the moment of the first sampling carried out on the corpse from which the analysis of the marker began.

In particular, 23 studies (82.1%) investigated the early PMI (interval 0–10 days from death), 7 studies (25%) included intermediate PMI (time between 10 days–1 month) and 6 studies (21.4%) evaluated late PMI (1 month–2 years).

#### 3.3.4. Type of Method Used in Human Studies

The most used method was Western blotting/immunoblotting (50%), followed by mass spectrometry (10.7%), immunohistochemistry (21.4%) and ELISA (7.1%). Other methods were used in 14.2% of human studies.

#### 3.3.5. Results Obtained in Human Studies

In 27 studies involving human models, the variation over time of at least one protein was observed. Only in one study was there no correlation between PMI and markers evaluated. The variations obtained were, in most cases, related to protein degradation or decrease (observed in 75% of the studies); in 5 studies (17.8%) there was an increase in at least one marker examined (Table 2).

### 3.4. Overall Analysis of Animal and Human Data

**Figure 2:**
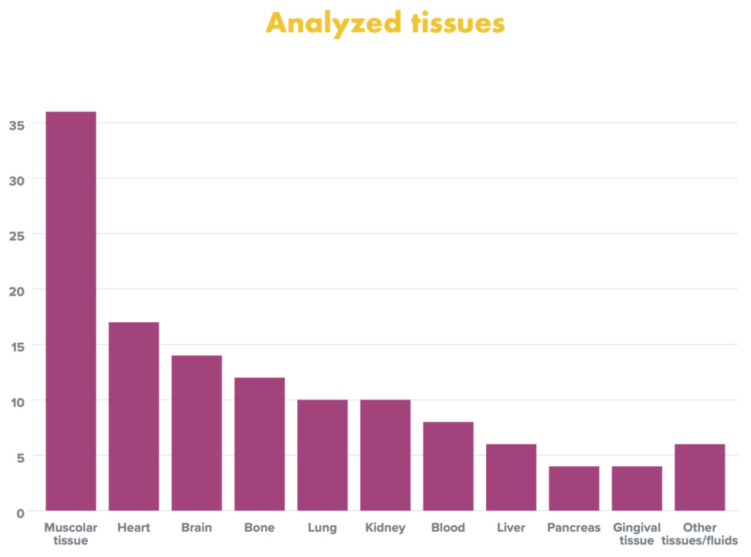
Overall frequency of tissue analysis examined in the studies selected for review (%).

Overall, the most analyzed tissues were: skeletal muscle tissue (36.1%), heart (17%), brain (14.8%), bone (12.7%), lung (10.6%), kidney (10.6%), blood (8.5%), liver (6.3%), pancreas (4.2%), gingival tissue (4.2%), spleen (2.1%), urine (2.1%) and cerebrospinal fluid (2.1%) (Figure 2).

**Figure 3:**
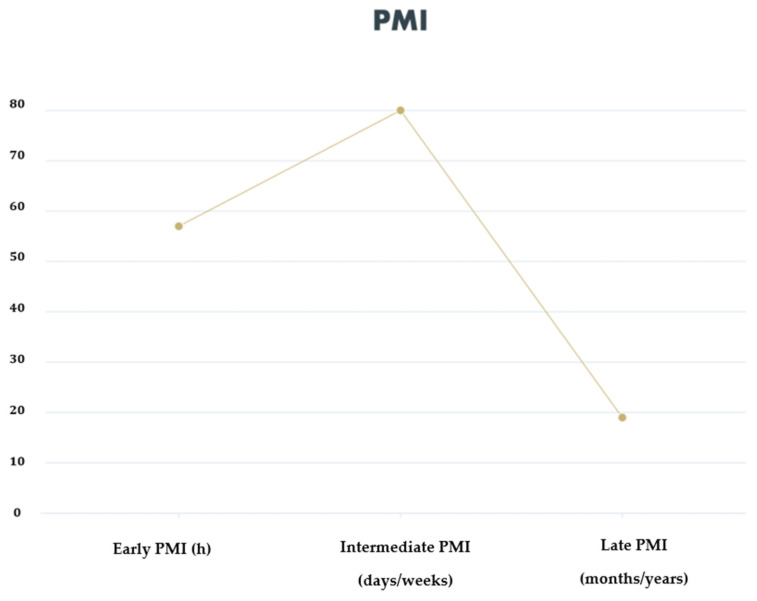
Overall percentage of studies that evaluated early, intermediate and late PMI.

The PMI included the analysis of the early PMI (0–24 h) in 27/47 studies (80.8%), analysis of the SME intermediary (1 day–1 month) in 38/47 studies (57.4%) and analysis of the late PMI (1 month–2 years) in 9/47 studies (19.1%) (Figure 3).

**Figure 4:**
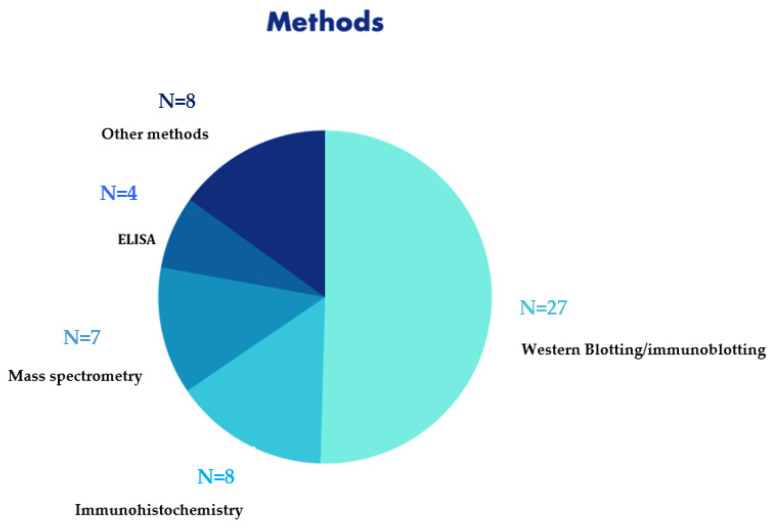
Overall frequency of analysis of the methodologies examined in the studies selected for the review (N=number of studies).

The most used methods were: Western blotting/immunoblotting in 27/47 (57.4%), mass spectrometry in 7/47 studies (14.8%), immunohistochemistry in 8/47 (17%), ELISA in 4/47 (8.5%) and other methods in 8/47 (17%) (Figure 4).

## 4. Discussion

The review of literature has shown that numerous post-mortem modifications of the proteome may occur. Each protein has an intrinsic stability, that is, the tendency to maintain its native structure. Proteins are typically very sensitive structures to temperature changes as they can cause denaturation with loss of functionality and loss of the native structure. The mechanisms plausibly involved in post-mortem stability, for a certain time range, may depend on various factors, including the biological sample chosen for the analysis, the environmental temperature (which affects the degradation kinetics), the cause of death (for example, the association between cardiac causes and influence on cardiac markers) and the method used for investigation, but also the onset of putrefaction (which involves changes in systemic pH and acid-base balance) [56,57,58,59,60,61,62,63,64,65,66,67,68,69,70,71,72,73,74,75]. With the same methods and sample analyzed, each protein shows a distinctive stability, for which the mechanisms possibly involved in the differences found in their post-mortem half-life may include its structure and its intermolecular interactions. The results show that the corpse cannot be considered only an organism that has ceased its vital activities, but as a system in continuous transformation, characterized by a complex biochemistry that varies over time. Starting from this assumption, numerous researchers have evaluated the possibility of examining post-mortem changes in protein levels as a function of time, with the aim of identifying a potential “proteic clock”, i.e., one or more PMI-related protein markers. The review showed a progressive interest in protein analysis technologies as a valid aid to forensic investigations on the time of death. More than half of the works published on this topic date back to the last five years (2016–2021)—evidence of a growing interest in this area of research.

### 4.1. Comparison of Experimental Models in the Literature

In the case studies examined, the authors proposed two main types of experimental model, the animal one and the human one. The most used animal models were with pigs and with rats or mice. The protocol in the animal model consisted of the extraction of a biological sample from the moment of the animal’s death and subsequent sampling at serial intervals. Certainly, the advantage of using animal samples is related to the possibility of knowing the exact moment of death, standardizing the causa mortis and exposing the corpse to precise temperatures. In humans, however, the analyses always began after the discovery of the body at the scene, therefore it is evident that the exact moment of death was not known to the authors and that the body could have been subjected to thermal variations before the autopsy, related to climatic factors or to the refrigeration of the body. Furthermore, the cause of death could vary from case to case, as well as the age of the subject and the comorbidities. Therefore, in human models performed in forensic cases, there was more inhomogeneity than in animal models. Different time frames were examined in each study. We have distinguished three main categories with the aim of making the data homogeneous, namely:

- Early PMI, i.e., a range that can be evaluated in hours (0–24 h from death);
- Intermediate PMI, which is a range that can be calculated in days or weeks (time between 1 day–1 month);
- Late PMI, i.e., a range that can be measured in months or years (1 month–2 years).

Based on this classification, various tissues were analyzed. In the early–intermediate PMI, the most analyzed tissue was the muscle. Instead, in the late PMI, the most examined tissue was bone. These findings are probably related to the stability of these tissues in the indicated times, i.e., days/weeks for the muscle and months/years for the bone.

### 4.2. Parameters Evaluated and Comparison of the Results in the Early–Intermediate PMI

Most of the studies were focused on early PMI and demonstrated a degradation or decrease pattern of proteins. This phenomenon can be explained on the basis of the autolysis beginning immediately after death, but also with self-destruction of tissues by the lysosomal proteolytic enzymes. Furthermore, considerable variations in pH and temperature occur after death, with progressive tissue destruction due to putrefaction, which certainly affect protein degradation in various ways depending on the characteristics and resistance of the single marker. Besides, the phenomenon of protein degradation/decrease is not identical for all proteins. Even within the same tissue, proteins travel at different degradation rates and some of them may have a higher rate of proteolysis than others that show more stability, even for days. The proteins showing the highest level of evidence to date are muscle proteins, including troponins. They are proteins used in clinical practice as indices of myocardial necrosis, being very susceptible to ischemic alterations [67]. Literature suggests that troponins may be useful markers for forensic purposes in early/intermediate PMI. Besides, they show a proteolytic degradation that is dependent on the time elapsed since death and occurs in both cardiac and skeletal muscle. The native protein progressively degrades over time, decreasing its concentration at specific time intervals. Furthermore, it gives rise to numerous minor fragments, characterized by a lower molecular weight than the original protein, each of which can be identified and quantified. The comparison of results in forensic human cases consisting of corpses found at various PMIs and exposed to various temperatures before their finding has shown a good correspondence with the results in animal experimental models created at a standard temperature.

**Figure 5:**
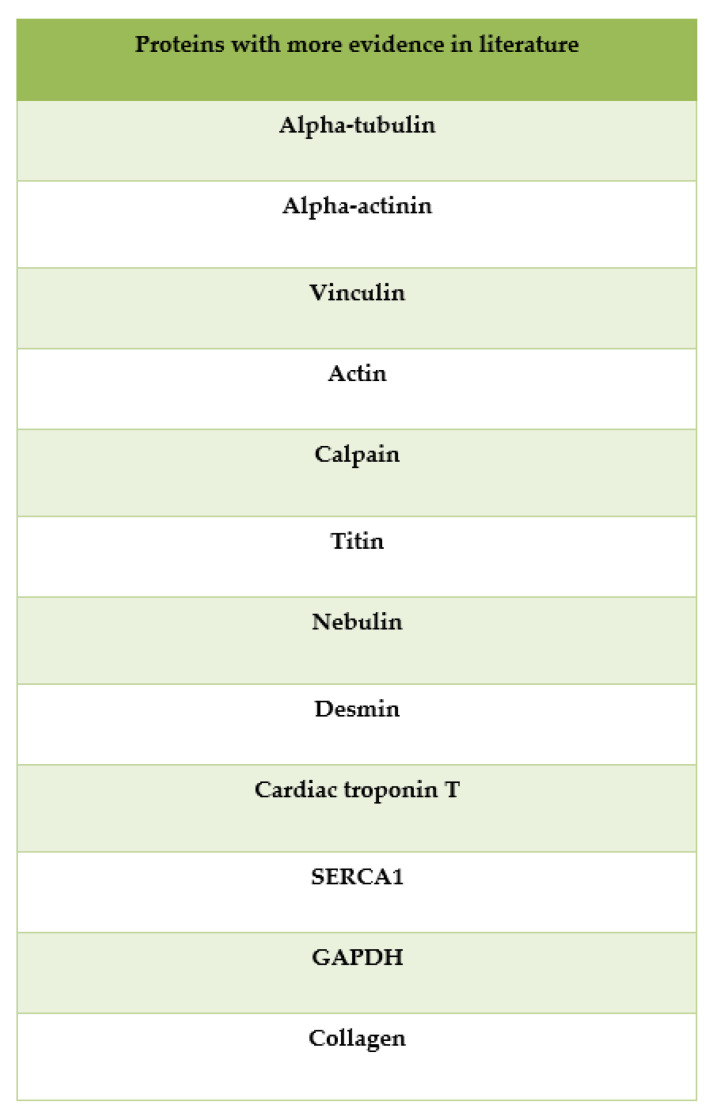
Markers that showed more evidence in the studies selected for review.

Many other proteins have also shown time-dependent fluctuations in skeletal muscle, such as, for example, alpha-tubulin, alpha-actinin, vinculin, actin, calpain, titin, nebulin, desmin, sarco-endoplasmic reticulum calcium ATPase (SERCA1) and glyceraldehyde 3-phosphate dehydrogenase (GAPDH), together with the related proteolytic fragments of lower molecular weight, whose appearance or disappearance may occur in specific post-mortem ranges [21]. Finally, the role of collagen should be noted, which has shown a decrease over time in various studies conducted on bone, gingival and kidney tissues, even with different methods (Figure 5).

All the other markers described in the tables and searched in other tissues or biological fluids in the early–intermediate PMI, despite having shown direct correlations with time, have not yet reached sufficient levels of evidence, i.e., a number of studies and cases analyzed adequate to predict its behavior with certainty and to recommend its systematic use (<2 papers). Therefore, we hope for an increase of studies on all the proteins described in the table that have shown time-dependent quantitative or qualitative variations. Among these, although with insufficient levels of evidence, there are also proteins that have shown an increase in early/intermediate PMI, such as tau, p-tau (in cerebrospinal fluid), high-mobility group box 1 (HMGB-1) (in blood) and glial fibrillary acidic protein (GFAP) (brain), whose variations could be related to the greater resistance to post-mortem changes and, therefore, to an intrinsic greater stability, or to a post-mortem synthesis.

The overall intersection of the data obtained from the variations of several markers, both in terms of increase and decrease, could be fundamental to estimate the PMI with increasingly precise intervals. Furthermore, we emphasize not only the need to precisely identify a certain range, but also to be able to exclude it. In this regard, interesting data emerge about the application of immune-histochemical staining on proteins expressed by organs such as the thyroid and pancreas. In a human study, somatostatin was shown to be stainable in the first ten days following death [61]. In another human study, insulin was shown to be detectable up to 18 days and glucagon up to 12 days after death. Regarding the thyroid, both thyroglobulin and calcitonin were stainable up to 8 days after death [39]. These results can be useful for a general overview of the time of death, as the positivity for one of these markers, in combination with other transformative signs of the body, can help to understand up to what days death may have occurred. Other immunohistochemical studies have been performed on gingival tissues, muscles and organs such as the heart, liver, kidneys and brain with similar results. A fundamental aspect of these investigations is, therefore, represented by the exclusion of certain time ranges. For example, in the study described above, if the immunostaining for insulin was found to be positive, the death certainly did not occur more than 18 days earlier. Unfortunately, it is not possible to have more detailed information regarding a specific range of hours in which death occurs, given that with immunostaining, the reference time unit is days and not hours. For this purpose, it is possible to evaluate other methods that have been used successfully. Among these, Western blotting has shown various advantages, such as low costs, ease of use and excellent evidence in the literature. Similar considerations apply to the ELISA technique, which has shown the possibility of quantifying the markers with low costs and rapid timing. Mass spectrometry, on the other hand, was mostly used for the analysis of bone samples in the late PMI. In only one study, the procedure was used in early PMI and evaluated muscle tissue samples.

### 4.3. Parameters Evaluated and Comparison of the Results in the Late PMI

Over the last few years, studies about late PMI have had an increase. Our review showed eight works, all published after 2017, involving experiments on samples of subjects that have been deceased for months or years. The investigations were focused on the analysis of the proteome, both on animal and human bones, by using mass spectrometry. The methodology used involved the burial of the subject with long-term sampling and subsequent analysis. Of course, the timing of these protocols is longer, and the lower number of published papers compared to the early PMI could be due to the complexity of the model and the related costs. In any case, the bone investigations on the late PMI have also shown excellent results, with identified protein changes. The results demonstrate the presence of continuous post-mortem protein modifications, even after years and in apparently stable tissues such as bone.

### 4.4. Limitations of Post-Mortem Protein Investigations

The review highlighted some technical limitations that will certainly represent the challenge for the next experiments:

- The temperature, which can alter the kinetics of protein degradation. We want to highlight the importance of these data as we believe it is necessary to focus research on proteins that have good resistance to thermal variations. Therefore, we believe it is crucial that the studies always analyze models with different sample exposure temperatures in order to evaluate, how much this variable could influence the kinetics of the studied protein;
- The effects of extrinsic variables, such as the cause of death, which can influence the variation of proteins such as troponins;
- Low levels of evidence related to the lack of statistical significance of the markers examined;
- Still missing evaluation of many tissues;
- The scarcity of data relating to biological fluids. We emphasize the importance of identifying markers in post-mortem fluids, such as blood, considering the easy sampling, even through an external body examination;
- The lack of human models examining “time 0”. All human models concerned corpses found at an unknown time after death, which were subjected to environmental variables or refrigeration. This limitation can affect the accuracy of the model on humans.

### 4.5. Proposals and Future Perspectives

This paper has shown how the study of proteins can be a valid aid to forensic investigations, in particular in cases where the time of death is decisive for evaluating circumstantial data, identifying suspects in specific time intervals and comparing the veracity of any testimonies [76].

**Figure 6:**
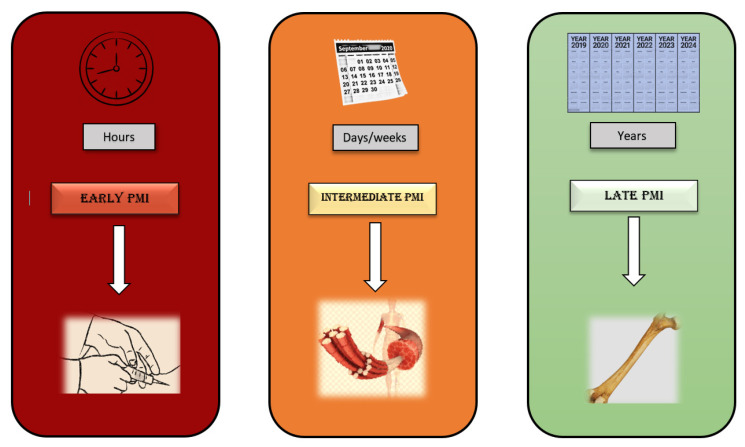
Operating protocol proposed in early PMI (analysis of biological fluids), intermediate PMI (muscle analysis) and late PMI (bone analysis).

From a technical point of view, the forensic pathologist finds greater difficulties in estimating the early PMI; in particular, the calculation of this time range would be useful for many forensic cases, especially when in homicides it could be necessary to determine the time of death precisely to investigate the role of possible suspects [74] (Figure 6).

The review and described limitations evidenced the need to standardize the human model by reducing possible biases due to intrinsic or extrinsic variables. For this purpose, we emphasize the possibility of including the evaluation of “time zero” in human models through the protocol proposed by Aquila et al. which involves the analysis of patients in hospital units with timely monitoring of vital parameters and vital functions through ECG [76]. This model would allow researchers to know exactly the moment of death, keeping the corpse at standard temperatures and examining biological samples with low invasive serial sampling on the body. We believe that biological fluids, and in particular blood, in a similar way to K+ in the vitreous humor, at least in the immediacy of death, i.e., before the onset of putrefaction, may be a useful sample for estimating the early PMI. This hypothesis, currently only partially verified, would allow a low-invasive analysis on the body. Instead, for the analysis of intermediate or late PMI, the most representative tissues, according to literature data, are muscle and bone due to their stability in these time ranges.

We hope that the progress of the research in the coming years will allow researchers to deepen the role of other protein markers, the comparison of which could allow us to build a mathematical model, with valid statistical significance for the calculation of the PMI on human samples, in view of a future rapid application of these methods in crime scene investigations.

## Figures and Tables

**Table 1:** Papers selected from the review on animal models, with analysis of the sample examined, number of cases, PMI investigated, method used, marker investigated and results. ↑ increase; ↓ decrease/degradation; - no correlation.

Authors	Animal	Sample	N. of Cases	Post-Mortem Interval (PMI) Evaluated for Analysis of Marker	Method	Protein Marker Investigated	Correlation of the Protein with Increasing PMI Analyzed	Result
Geissenberger J et al., 2021 [10]	Pigs	Skeletal muscle	6	0–240 h	Western blotting	Alpha-tubulin Alpha-actinin, GAPDH Vinculin	Degradation	↓
						Tropomyosin	Stability	-
Wang J et al., 2021 [11]	Mice	Skeletal muscle	60	0–96 h	Western blotting	PP2A-B P-PP2A-C (Tyr-307)	Degradation	↓
						PP2A-C	Stability	-
Welson NN et al., 2021 [12]	Rats	Myocardium Kidney Testes	42	0–120 h	Tissue levels measurement	Malonaldehyde (MDA),	Increase	↑
						Superoxide dismutase (SOD) Reduced glutathione (GSH)	Decrease	↓
					Immunohistochemical staining	B cell lymphoma 2 (BCL2)	Staining reduction	↓
Zhang Y et al., 2020 [13]	Rats	Serum	54	6–168 h	ELISA	TN-T VEGF HIF-1α	VEGF/HIF-1α showed a significant relation with PMI	
Pittner S et al., 2020 [14]	Pigs	Skeletal muscle	8	0–14 days	Western blotting	Cardiac troponin T Vinculin Desmin	Degradation	↓
						Tropomyosin	Stability	-
Choi KM et al., 2019 [15]	Rats and mice	Skeletal muscle	25	0–96 h	LC/MS–MS analysis Western blotting	GAPDH eEF1A2	Decrease and degradation	↓
Procopio et al., 2018 [16]	Pigs	Bone	4	1 month-1 year	LC/MS–MS analysis	Bone proteome	Variations in the decay rate of several proteins	
Ehrenfellner et al., 2017 [17]	Pigs and mice	Muscle tissues	3	0–10 days	Western blotting	Alpha actinin, Alpha tubulin, Fast skeletal muscle troponin T Vinculin, Desmin, Cardiac troponin T	Degradation	↓
						Tropomyosin	Stability	-
Procopio et al., 2017 [18]	Pigs	Bone	5	12 days–24 months	LC/MS–MS analysis	Alpha-1 antitrypsin Chromogranin-A	Increase	↑
						Fetuin-A	Decrease	↓
Hahor et al., 2016 [19]	Fishes	Gastrointestinal tracts	-	0–48 h	Specific activity Assays	Pepsin activity Trypsin activity Chymotrypsin activity Amylase activity Lipase activity	Decrease	↓
					Protein measurement with the Folin phenol reagent	Stomach and intestinal protein concentrations in the crude enzyme extracts		
Lee et al., 2016 [20]	Rats	Kidney Skeletal muscle	48	0–96 h	Western blotting Immunohistochemistry	Glycogen synthase (GS) Glycogen synthase kinase-3β AMP-activated protein kinase α Caspase 3 Glyceraldehyde 3-phosphate dehydrogenase (GAPDH)	Degradation	↓
						p53 β-catenin	Stability	-
Pittner et al., 2016 [21]	Pigs	Skeletal muscle	3	0–240 h	Western blotting	Titin Nebulin Desmin Cardiac troponin T SERCA1 Calpain	Degradation	↓
						Tropomyosin α-actinin	Stability	-
Foditsch et al., 2016 [22]	Pigs	Skeletal muscle	2	0–21 days	Western blotting SDS-PAGE gel analyses	Desmin Nebulin Titin SERCA 1 μ-calpain	Degradation	↓
						α-actinin Calsequestrin-1 Laminin Troponin T-C SERCA 2	Stability	-
Boaks et al., 2014 [23]	Pigs	Bone	5	0–12 months	Spectrophotometry	Co/NCo proteins (collagenous and non-collagenous)	Reduction	↓
Kikuchi et al., 2010 [24]	Rats	Blood	90	0–7 days	ELISA	HMGB-1	Increase	↑
Poloz et al., 2009 [25]	Mice	Skeletal muscle	4	0–96 h	Western blotting	CnA	Degradation	↓
						PP2A CaMKII	Reduction	↓
		Lung				MARCKS PP2A	Reduction	↓
Curcio et al., 2006 [26]	Rats	Brain	-	4 h	Western blotting	Bag 1	No correlation	X
Sabucedo et al., 2003 [27]	Bovines	Myocardium	3	0–6 days	Western blotting	Intact cTnI degraded	Reduction	↓
Kang et al., 2003 [28]	Rats	Brain Lung Heart Kidney Liver Skeletal muscle Spleen	16	0–96 h	Calmodulin binding overlay technique (CaMBOT)	Calmodulin (CaM) binding proteins (CaMBPs)	No correlation	X
		Lung Muscle			Western blotting	Ca2+/CaM-dependent kinase II (CaMKII)	No correlation	X
						Calcineurin A (CNA)	Degradation	↓
		Lung			Western blotting	Myristoylated alanine-rich C-kinase substrate (MARCKS)	Reduction	↓
						Inducible nitric oxide synthase (iNOS)	No correlation	X

**Table 2:** Papers selected from the review on human models, with analysis of the sample examined, number of cases, PMI investigated, method used, marker investigated and related results. ↑ increase; ↓ decrease/degradation; - no correlation.

Authors and Year of Publication	Sample	Number of Cases Examined	Post-Mortem Interval (PMI) Evaluated for Analysis of Marker	Method	Marker Analysed	Correlation of the Protein with Increasing PMI	Result
Peyron PA et al., 2021 [29]	Cerebrospinal fluid	82	2.0–11.8 h	ELISA	Tau p-tau	Increase	↑
Mickleburgh HL et al., 2021 [30]	Bone	4	Date of burial-3 years after burial	LC/MS–MS analysis	Complement C3 collagen alpha-1(III) chain (CO3A1) Complement C9 (CO9) Collagen alpha-2(XI) chain (COBA 2) Matrix Gla protein (MGP) Decorin (PGS2) Transthyretin (TTHY)	Decrease	↓
Hu B.-J., 2020 [31]	Myocardium	5	1–28 days	Immunohistochemistry	Desmin Actin Myoglobin	Staining reduction	↓
Pittner S et al., 2020 [32]	Skeletal muscle	2	Date of burial-105 days after burial	Western blotting	Tropomyosin GAPDH eEF1A2	Decrease	↓
					Alpha-tubulin Alpha-actinin Vinculin	Degradation and decrease	↓
Pittner S et al., 2020 [33]	Skeletal muscle	3	2.4–42 days	Western blotting	Alpha-tubulin Alpha-actinin Vinculin	Degradation	↓
Mazzotti MC et al., 2019 [34]	Gingival tissues	10	3–9 days	Immunohistochemistry	Collagen type I protein Collagen type III protein	Staining reduction	↓
Choi KM et al., 2019 [15]	Skeletal muscle	3	15–>336 h	Western Blotting	GAPDH eEF1A2	Degradation	↓
Prieto-Bonete G et al., 2019 [35]	Bone	40	5–20 years	LC/MS–MS analysis	275 proteins	Specific proteins have been identified in different PMI	
Lesnikova et al., 2018 [36]	Liver Lung Brain	40	1–>14 days	Immunohistochemistry	Vimentin S100 PCK CD45	Staining reduction	↓
Fais et al., 2018 [37]	Gingival tissues	10	1–8 days	Immunohistochemistry	Hypoxia inducible factor (HIF-1α)	Decrease	↓
Pérez-Martínez et al., 2017 [38]	Bone	80	5–47 years	HPLC/MS/MS	Collagen type I protein	Decrease	↓
Ehrenfellner et al., 2017 [17]	Skeletal muscle	3	0.5–40 days	Western blotting	Alpha actinin Alpha tubulin Fast skeletal muscle troponin T Vinculin Desmin Cardiac troponin T	Degradation	↓
					Tropomyosin	Stability	-
Ortmann et al., 2017 [39]	Pancreas	105	Several h—22 days	Immunohistochemistry	Insulin Glucagon Thyreoglobulin	Staining reduction	↓
	Thyroid				Calcitonin		
Pittner et al., 2017 [40]	Skeletal muscle	2	-	Western blotting	Desmin Cardiac troponin T (cTnT) Calpain	Degradation	↓
					Tropomyosin	Stability	-
Campell et al., 2016 [41]	Brain	16	6–72 h	Immunoblotting	Talin	Decrease	↓
Blair et al., 2016 [42]	Brain	2	4.5–48 h	Western blotting	Alpha tubulin	Decrease	↓
					β-actin GAPDH PHF1 AT8 Tau-5	No correlation	X
					NeuN	Decrease (not in all examined cases)	↓
		6		Immunohistochemistry	GFAP Collagen COX-1 PHF1 AT8 Collagen IV	No correlation	X
					Alpha tubulin	Staining reduction (not in all examined cases)	↓
Kumar et al., 2016 [43,44]	Myocardium	60	5–230 h	Western blotting	Cardiac troponin-T	Degradation	↓
Pittner et al., 2016 [45]	Skeletal muscle	40	3.5–92.8 h	Western blotting Zymography	Tropomyosin	Stability	-
					Cardiac troponin-T Desmin Calpain	Degradation	↓
Kumar et al., 2016 [46]	Myocardium	6	15–189 h	Western blotting	Cardiac troponin-T	Degradation	↓
Kumar et al., 2015 [47]	Myocardium	5	5–230 h	Western blotting	Cardiac troponin-T	Degradation	↓
Kumar et al., 2015 [48]	Myocardium	9	8–88.4 h	Western blotting	Cardiac troponin-T	Degradation	↓
Sinha et al., 2012 [49]	Myocardium Pancreas Brain Lungs Liver Kidney	20	0–10 days	SDS-PAGE analysis	Transferrin Albumin Alpha-1 antitrypsin Haptoglobulin Glyceraldehyde dehydrogenase Glutathione S-transferase Hemoglobin subunits alpha and beta	Degradation	↓
Chandana et al., 2009 [50]	Brain	9	4–18 h	Western blotting	GFAP Synatophysin (SP) Neurofilament (NF)	Increase No correlation Increase	↑ X ↑
Kasuda et al., 2009 [51]	Urine	44	6–48 h	ELISA	von Willebrand factor	Increase	↑
	Blood					No correlation	X
Tavichakor-ntrakool et al., 2008 [52]	Skeletal muscle	1	1.4–48 h	Q-TOF MS/MS	Heat shock protein 27	Reduction	↓
					Myoglobin	No correlation	X
					M. creatine kinase	Increase	↑
				LDH assay	LDH activity	Increase	↑
Uemura et al., 2008 [53]	Blood	164	0–72 h	Latex aggregation method	HbA1c	No correlation	X
				Rate assay	C-reactive protein		
				Biuret method	Pseudocholine esterase		
				JSCC standardization method	t-Protein ɣ-GTP		
Crecelius et al., 2008 [54]	Brain	3	2 h (after autopsy)–48 h	Western blotting 2-D DIGE	Peroxiredoxin 1 Stathmin	Reduction	↓
					GFAP	Increase	↑
Thaik-Oo et al., 2002 [55]	Brain	19	1–120 h	-	Vascular endothelial growth factor (VEGF)	Decrease (after 40 h)	↓
	Lungs Kidneys					Decrease (after 24 h)	↓
	Heart					No correlation	X

## Data Availability

Not applicable to this article as no datasets were generated.
